# Engaging individuals in digital health research panels: A qualitative study including participants in vulnerable positions

**DOI:** 10.1371/journal.pdig.0001443

**Published:** 2026-05-22

**Authors:** Corine Oldhoff-Nuijsink, Mirjam P. Fransen, Jeanine Suurmond, Linda W.P. Peute, Marloes E. Derksen

**Affiliations:** 1 Department of Medical Informatics, eHealth Living & Learning Lab Amsterdam, Amsterdam UMC, University of Amsterdam, Meibergdreef, Amsterdam, The Netherlands; 2 Amsterdam Public Health, Digital Health, Amsterdam, The Netherlands; 3 Department of Public and Occupational Health, Amsterdam UMC, University of Amsterdam, de Boelelaan, Amsterdam, The Netherlands; 4 Centre for Communicable Disease Control, National Institute for Public Health and the Environment, Antonie van Leeuwenhoeklaan, Bilthoven, The Netherlands; 5 Radboud UMC, Department of Primary and Community Care, Geert Grooteplein, Nijmegen, The Netherlands; Shahid Beheshti University of Medical Sciences School of Dentistry, IRAN, ISLAMIC REPUBLIC OF

## Abstract

Individuals in vulnerable circumstances often face challenges in accessing and utilizing digital health tools. They are also underrepresented in digital health research. Consequently, digital health tools may not be aligned with their specific needs and requirements, potentially intensifying health disparities. A research panel especially involving individuals in a vulnerable position could increase their representation in digital health research. This study examined motivation, facilitators and barriers, and prerequisites for initial participation and sustained involvement in a panel for digital health research. We conducted 23 semi-structured interviews with mainly individuals in vulnerable positions. Interviews were audio recorded, transcribed, coded and thematically analysed. The results indicated that most participants were motivated for engagement in a research panel on digital health. They aspired to contribute to the accessibility and usability of digital health, thereby benefiting not only themselves but also their peers. Additional motivations were to stay informed and to learn from developments in the field. Participants perceived recruitment efforts in community centres or the distribution of inclusively designed flyers as the most effective methods. Clear communication and timely feedback of results emerged as the most significant factors in sustaining long-term enthusiasm for panel participation. As the study was conducted in the Dutch context, cultural and contextual factors may limit the generalisability of the results to other settings. Nonetheless, by fostering intrinsic motivation, tailoring recruitment approaches, and addressing practical barriers, research panels can facilitate meaningful participation and contribute to the equitable development of digital health interventions.

## Author summary

In this study, we investigated how individuals in vulnerable circumstances can be more meaningfully involved in research projects on digital health. Most of these individuals face difficulties in using digital health tools. Additionally, they are rarely included in research through which these tools are developed, resulting in tools that may not meet their needs and could even widen existing health gaps. We spoke with 23 people, most of whom were in vulnerable situations, to understand what motivates them to join a research panel, what helps or hinders their participation, and what is needed to keep them engaged over time.

We found that many individuals were eager to contribute to improving digital health tools, not just for themselves, but also for others in similar situations. They wanted to stay informed and learn more about developments in the field of digital health. Recruitment efforts that took place in familiar community centres and the use of accessible materials were seen as most effective. Participants also emphasized the importance of clear communication and receiving feedback about the impact of their involvement. Our findings suggest that inclusive recruitment and ongoing support are key to ensure that digital health research reflects the voices of those who are generally left out.

## Background and significance

The field of digital health is a promising domain that leverages digital tools to enhance healthcare delivery and management. Digital health includes all types of information and communication tools [1] (e.g., telemedicine platforms, wearable devices, mobile health apps or chatbots). These tools have the potential to make healthcare more effective, more personalized, and more accessible to a broader range of individuals [2,3]. As these tools become more integrated into healthcare, health professionals increasingly expect health consumers to be able to use them. For example, physicians may ask patients to monitor their glucose levels using a mobile health application.

However, these technological developments present challenges. One major worry is that digital health can unintentionally maintain or increase health inequities across different populations in society [4–7]. The digital divide is a well-known phenomenon that is defined by differences in access to, use of, and competence with digital technology [8,9]. This means that individuals in vulnerable positions – such as those with low socio-economic positions, low educational levels, minority backgrounds, elderly or people living in rural areas – are more likely to have difficulty accessing and utilizing digital tools [8,10]. Challenges among these individuals could include the lack of adequate digital health literacy or the unavailability of appropriate technological devices. The World Health Organization defines digital health literacy as the ability to search, find, understand and evaluate health information from electronic resources and apply the knowledge gained to address or solve a health problem [11].

In addition to the fact that individuals in vulnerable positions have difficulty using digital health tools, they are also underrepresented in scientific research [12–14]. One of the arguments is that these groups are generally harder-to-reach with passive recruitment methods, such as a flyer [15,16]. In general, researchers have too little time and budget for active recruitment and/or limit themselves to groups that respond first and are therefore relatively easy to reach. On the other hand, individuals in a vulnerable position can have relatively more difficulties understanding research information, understanding consent forms, or providing consent, may experience low self-efficacy [14,17] or hold beliefs that participating in research does not benefits the community [18]. Underrepresentation of people in vulnerable positions in research may lead to development and implementation of digital health tools that are only accessible to and effective among the general population [19]. If they do not participate in development process, the expectations, needs and requirements of individuals in a vulnerable position (mental models) are not reflected in the design of digital tools (conceptual models). Consequently, tools are not utilized by all intended end users, wasting invested time and money [20,21], and leading to health inequities [6].

It is crucial to involve representative end users in the development, implementation, and evaluation of digital health tools [22,23], for example, by using the User Centered Design principles [24]. Literature indicates that involving end users throughout the development of digital health tools helps identify unmet needs, improves usability and desirability, and enhances adoption and long-term use. These factors contribute to better health outcomes and ensure that technology fits better into the overall patient journey [25,26]. Although, some studies describe that end users do get involved in the development of such tools, it is mainly in later stages of the research cycle, such as testing a prototype. They are less involved in earlier phases of the research, such as identifying end user needs and translating them into design considerations. Also, participants are often seen as test subjects and not as equal partners, having less to say than other stakeholders within the research project [27]. It is important to incorporate insights, skills, needs and preferences of a diverse group of end users [28], leading to more inclusive designs, improving equity and access to healthcare [26].

One possible solution to engage a representative study population is panel research. A research panel is a fixed group of motivated and representative individuals. Panel members participate in multiple research projects over time, for example, in co-creation sessions or usability studies on mobile health apps. This repeated engagement allows researchers to build trust, tailor communication, and reduce the effort of repeated recruitment. Fairbrother et al. [29], for example, showed that a clinical patient panel for home telemonitoring fostered greater openness and collaboration. At the same time, panels are not without their limitations. While they can lower participation barriers, they may also reproduce inequities. For example, if recruitment favours those feeling confident expressing their perspectives and concerns, thereby unintentionally excluding more introverted people who may nonetheless offer valuable insights. It may also marginalise those who feel less competent with digital tools, resulting in a loss of perspectives from individuals who are less digitally skilled and potentially more concerned about the developments of digital health services.

However, there is currently limited knowledge on how to recruit and maintain the involvement of vulnerable groups in research panels on digital health. Therefore, we aimed to gain more insights into the perspectives of individuals, with a particular focus on those in vulnerable positions, on their motivations, facilitators, barriers and conditions influencing initial participation and long-term engagement in a panel focused on digital health research.

## Methods

We performed semi-structured qualitative interviews. We followed the COREQ criteria for reporting this study [30].

### Ethics statement

Following Dutch law, the Medical Ethics Review Committee of Amsterdam UMC, approved this non WMO study (number: 2023.0289). We ensured that all participants understood the purpose of this study and guaranteed that their participation was voluntary and pseudomized. All participants provided written informed consent prior to enrolment in the study.

### Participants and recruitment

**Fig 1:**
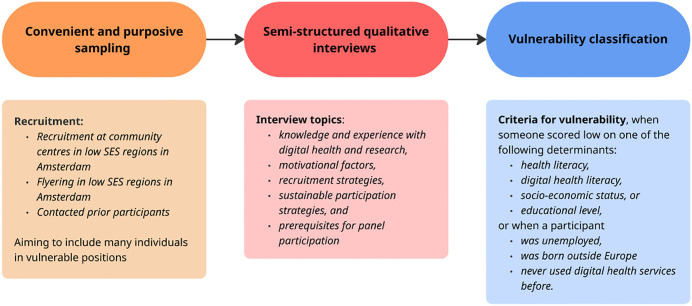
Key procedures.

We applied convenient and purposive sampling, aiming to primarily include individuals in vulnerable positions (see Fig 1). We classified participants as being in a vulnerable position when they scored low on at least one of the following criteria: health literacy, digital health literacy, socio-economic position (postal code score), educational level, or when someone was unemployed, being born outside Europe, or never used digital health services before.

Health literacy was measured with the NVS-D instrument [31], with scores three or lower classified as low. Digital health literacy was measured with a shortened version of the eHeals (items 1,4,7,8) [32]. Because no validated cut-offs exist for this abbreviated scale, we classified participants as having low digital health literacy when they responded “strongly disagree” or “disagree” to at least two of the four questions. Socio-economic position, employment status and educational level are based on predefined criteria of Statistics Netherlands [33]. Socio-economic position was based on postal code scores. Employment status was determined on the basis of employment in the last four years, classified as either employed or not employed. Educational level was measured using the ISCED classification, participants were classified as having a low educational level when their highest educational attainment was primary education, prevocational secondary education, years 1–3 of higher secondary education, or vocational secondary education. We also asked participants if they have ever used digital health services before (this could be any form, including using patient portals, health apps, wearables, or even simple online searches for health information such as googling symptoms). This broad operationalisation of vulnerability enabled us to include a diverse group with varied perspectives, supporting a rich exploration of experiences and viewpoints related to a panel for digital health research.

To achieve this, two research assistants (TK, MC) actively recruited individuals in community centres in low SES areas in Amsterdam. We also invited individuals in vulnerable positions that had participated in our earlier research and had indicated they were willing to participate in other research projects, via phone calls or email. One reminder mail was sent to individuals who had not replied within one week. Because this approach relied on community-based recruitment and outreach to prior participants, it may have led to an oversampling of individuals who were already open to research participation. This could have increased the likelihood of positive attitudes toward joining a panel and may have influenced the themes that emerged from the analysis. We therefore interpreted participants’ expressed willingness and motivations with this sampling context in mind.

The passive approach included strategies such as placing flyers (S2 Flyer) in local stores, hospitals, healthcare and community centres in low SES areas in Amsterdam and sharing the flyer on specific social media pages (e.g., Facebook groups focused on people experiencing poverty). Although we recruited at locations predominantly visited by individuals in vulnerable positions, we did not exclude participants upon registration to explore panel participation amongst a diverse population.

Individuals who indicated an interest in participating were informed by an information letter about the study, received by e-mail two weeks before the interview took place. Before starting the interview, participants were informed that the study aimed to obtain an understanding of the motivations, facilitators and barriers and prerequisites for participating in a research panel on digital health.

### Data collection

Data were collected by qualitative interviews held in November 2023 (conducted by a female researcher CON (MSc in Health Sciences, experienced in interviewing)) and August 2024 (conducted by a female researcher MC (MSc in Medicine, received interviewing training prior to interviews)) (see Fig 1). The interviews were conducted at a place the participant preferred, participants’ homes (n = 2), in an academic hospital (i.e., Amsterdam UMC, location AMC, (n = 10) or online via Microsoft Teams (n = 11). Interviews were in Dutch, one-off and lasted about 45 minutes. Participants were rewarded with a €20 gift card and travel and parking expenses were reimbursed if applicable. Participants received incentives to reimburse time and expenses to reduce participation barriers; incentives were framed as acknowledgements rather than to externally motivate them to participate. Researchers had no relationship established with participants prior to the study. During the interviews, no one else was present besides the participant and the researcher(s).

To ensure consistency across interviewers and data collection waves, MC was present during several interviews conducted by CON. Both interviewers used the same interview guide, and they regularly discussed interview techniques to maintain a consistent approach. Although interviews were conducted at different locations and times, we did not observe systematic differences in themes across interviewers, waves or settings.

Interview questions were pilot tested with a colleague to assess clarity of the questions, length of the interview and whether relevant questions were missing. During the interview, we assessed participants’ background characteristics. Relevant participant characteristics are presented alongside each quotation in the result section.

The interviews were audio recorded, and field notes were made by CON or MC. A semi-structured interview guide (S1 File) was used, based on knowledge from previous research as well as grey literature on the challenges and facilitators of research panels. Topics covered in the interview guide included current knowledge and experience with digital health services and scientific research (panels), motivational factors, recruitment strategies, sustainable participation strategies, and prerequisites for a research panel. During the interviews, interview cards (S3 File) were used for visual support.

### Data analysis

The raw interview data were transcribed verbatim by CON and MC. Transcripts were not sent to participants for comments, as this option was not included in the informed consent form. Data were coded and analysed in MAXQDA (2022). In the initial round of coding, CON and MD independently coded three randomly selected interview transcripts. Disagreements between MD and CON were discussed in a consensus session. No major differences found during the coding process, discrepancies mainly concerned the level of detail and length of the codes. CON coded all remaining transcripts. We assessed data saturation by checking whether new transcripts generated codes that required additions or changes to the developed coding tree. A “new theme” was defined as a code that introduced a new idea, rather than simply providing another example of an existing theme. After the interviews in November 2023, the authors agreed that data saturation has not been reached. Therefore additional interviews were conducted in August 2024, and data saturation was considered reached. Regular research meetings were held to foster reflexivity in data analysis.

Thematic analysis was employed to systematically examine the interview data. Following Braun and Clarke’s (2006) six-phase framework [34], the process began with familiarization with the data through repeated reading of the interview transcripts. An inductive coding approach was then applied; codes were iteratively refined and organized into a structured coding tree, which supported the development of themes. These codes were organized into potential themes, which were reviewed and refined to ensure they accurately captured the nuances of the data. Theme decisions were made collaboratively during research meetings, where different interpretations of coded text were discussed. These decisions were not systematically documented but were reached through team consensus. Reflexivity was actively integrated into the data analysis. Beyond regular research meetings, each coder reflected on their assumptions about panel participation and expectations that might unintentionally influence the interpretation of what was meant by participants. We also considered how our role as researchers, and the inherent power dynamics in interviewing, might have shaped what we prioritised in the data. These reflections were revisited during the refinement of themes to ensure that interpretations remained grounded in the data rather than in researchers’ preconceptions.

Throughout the analysis, a rigorous approach was maintained by regularly cross-referencing the themes with the raw data to enhance validity. The final themes were defined and named to provide a comprehensive understanding of participants’ perspectives and experiences. Participants did not provide feedback on the findings, as this was not included in the informed consent form.

#### Development of research panel.

First, we conducted the interview study, thereafter we established a research panel focused on digital health projects. Insights derived from this interview study informed the design and operational structure of the panel.

## Results

### Participant characteristics

In this study 23 individuals participated (see Table 1). More females (n = 18) than males (n = 5) participated, the average age was 54 year (range 24–76). About 75% (n = 17) of our participants were in a vulnerable position. Most of them (n = 15) scored low on multiple ‘vulnerability’ determinants.

**Table 1:** Background characteristics of participants n = 23.

ID	Participants	Age	Work status	Born in	Education	SES	Health Literacy	Digital Health Literacy	Ever used digital health services
	Females n = 18 Males n = 5	Mean =54	Employed n = 7 Unemployed n = 16	Netherlands n = 13 Outside Europe n = 9 Inside Europe n = 1	Low n = 3 Medium n = 12 High n = 8	Low n = 11 Medium n = 4 High n = 7	Low n = 11 High n = 12	Low n = 5 Medium n = 6 High n = 12	Yes n = 14 No n = 9
#1	*Female*	*28*	*Unemployed*	*Netherlands*	*Medium*	*Low*	*High*	*High*	*Yes*
#2	*Female*	*65*	*Unemployed*	*Outside Europe*	*Low*	*Low*	*Low*	*Low*	*No*
#3	*Female*	*66*	*Unemployed*	*Outside Europe*	*High*	*Low*	*High*	*High*	*No*
#4	*Male*	*67*	*Unemployed*	*Netherlands*	*High*	*Low*	*High*	*Medium*	*No*
#5	*Female*	*29*	*Unemployed*	*Netherlands*	*Medium*	*High*	*High*	*High*	*Yes*
#6	*Female*	*54*	*Unemployed*	*Outside Europe*	*Medium*	*Medium*	*High*	*High*	*Yes*
#7	*Male*	*51*	*Unemployed*	*Netherlands*	*Medium*	*Low*	*Low*	*Medium*	*Yes*
#8	*Female*	*57*	*Employed*	*Netherlands*	*Medium*	*High*	*Low*	*High*	*Yes*
#9	*Female*	*50*	*Unemployed*	*Netherlands*	*Medium*	*High*	*Low*	*Low*	*Yes*
#10	*Female*	*41*	*Unemployed*	*Netherlands*	*Medium*	*High*	*High*	*Medium*	*Yes*
#11	*Female*	*41*	*Unemployed*	*Netherlands*	*Medium*	*Low*	*High*	*Medium*	*No*
#12	*Female*	*36*	*Employed*	*Inside Europe*	*Medium*	N/A*	*High*	*High*	*Yes*
#13	*Female*	*58*	*Employed*	*Netherlands*	*High*	*Medium*	*High*	*Low*	*No*
#14	*Female*	*58*	*Employed*	*Outside Europe*	*High*	*Low*	*Low*	*High*	*Yes*
#15	*Male*	*69*	*Unemployed*	*Outside Europe*	*Low*	*Low*	*High*	*Medium*	*No*
#16	*Male*	*63*	*Employed*	*Netherlands*	*Medium*	*High*	*High*	*High*	*Yes*
#17	*Female*	*51*	*Unemployed*	*Netherlands*	*High*	*Medium*	*High*	*High*	*Yes*
#18	*Female*	*58*	*Unemployed*	*Outside Europe*	*High*	*Low*	*Low*	*High*	*Yes*
#19	*Female*	*24*	*Employed*	*Netherlands*	*High*	*Low*	*Low*	*High*	*Yes*
#20	*Female*	*59*	*Employed*	*Netherlands*	*Medium*	*High*	*Low*	*Medium*	*Yes*
#21	*Male*	*76*	*Unemployed*	*Netherlands*	*High*	*Low*	*Low*	*Low*	*No*
#22	*Female*	*74*	*Unemployed*	*Netherlands*	*Low*	*Medium*	*Low*	*High*	*No*
#23	*Female*	*64*	*Unemployed*	*Outside Europe*	*Medium*	*High*	*Low*	*Low*	*No*

## 1. Motivation for participation in digital health research

The *Motivation* theme comprises factors that participants regard as relevant when deciding whether to participate in a research panel on digital health. Almost all participants were enthusiastic and motivated to become panel member, reflecting a variety of motivations.

### Contributing to research and having a voice being acknowledged

Some participants derived considerable satisfaction from contributing their insights and experiences to digital health research. They expressed appreciation for the opportunity to share their perspectives and experiences and valued the recognition by researchers of their input as critical. What motivates them was if their input was translated into health policy or practice.

*For me, it’s because I work in healthcare [...] I really enjoy contributing to such studies. I enjoy sharing my experience and knowledge.* P19

*The reason I participated in this interview was for me the curiosity of yes tell my story. But I am also happy when I know that it is going to end well, so that the person is listening to me. That is more important to me.* P23

### Help others

Participants generally indicated that they wanted to participate in order to contribute to society, or to help other individuals. They saw their participation in research as a way to help others who are in similar situations. They argued that their motivation was frequently linked to personal experiences, they expressed empathy for others facing similar challenges regarding digital health. They believed that their contribution could lead to the development of more accessible digital health solutions, for them self and others in their community and beyond.

“*That is also the motive for participating in surveys. I also enjoy being able to help my fellow man*.” P8

“[...] *I had heart failure. Well maybe still do. But yes, but then when you look up that info, you get the info, you’re going to die 50% chance within one to two years. And I find like this, I actually don’t think it’s okay, because It’s not like this [prognosis] anymore. This was maybe 10 years ago, but right now [...]it is catching up, so this [online information] is not up to date anymore and I also don’t think it’s positive, because I passed that two years and I’m probably going to, hopefully I’m going to pass the coming five and ten years as well. But it immediately gives such a stamp If you. Yes, you’re going to die, But you really don’t want to [hear] that at that time. It made me very depressed. [...] Why do I get things like that and not good information?”* P20

### Societal contribution

Others gave the reason that since they were no longer working (e.g., retirement) or able to work, they still wanted to contribute to society. They saw participating in research projects on digital health as a low-threshold way to achieve this.

“*Because I don’t work anymore, let me put it this way, yes, you are more open to this anyway. Because A you have the time and B yes, you do want to feel a bit useful*.” P7

### Influence on digital health developments

Some participants were driven by a desire to learn more about digital health and how it could potentially impact their daily lives. They exhibited interest and curiosity in comprehending most recent developments in the field and how these could contribute to a healthcare process. This curiosity often extended beyond their personal situation, with participants expressing a general interest in digital health innovations. They were eager to learn about developments, viewing their participation in the research panel as a unique opportunity to satisfy this curiosity. Additionally, some participants expressed their concerns about the rapid advancements in digital health or concerns about control external parties may have through digital technologies, as outlined as follows:

*“I find it a bit scary, but I am curious, yes you have to participate somehow. I don’t think citizens have that much to say. Because one [government] just does it. For example, with debit cards, I put off debit cards for as long as I could. But almost everywhere you have to use debit cards now. The government wants to control you so yes, you know, on the one hand it’s easy. On the other hand, they can check all your movements. They really know everything about you. You can’t get out of it.”* P2

*“Yes, I am kind of curious of what’s to come in that area. Also, of yes what else is possible, which maybe I don’t know at all. I just find it interesting of yes, what else is coming in the future, shall we say.” P13*

Additionally, it was noted that the pace of digital advancements is so rapid that participants wished to stay abreast of these changes and contemplate their implications to ensure that digital health remains practical and accessible.

*“Learning always yes. Gaining insight both into the case being put in front of me, but also insight into yourself of course. Because you have to think about things you might not actually think about so quickly, and I like that too.”* P7

Not all participants’ harboured enthusiasm towards digital health. A number of individuals indicated that they found it more pleasant to maintain physical contact with a healthcare provider as much as possible. They explained that they did not feel comfortable to arrange their health matters independently or were afraid that the quality of care will decrease if they have to manage it themselves. However, even among those who harboured reservations, there was a recognition that the cessation of hybrid/digital health is implausible, and that the era of exclusively physical care has passed. Thus, they felt compelled to adapt to hybrid/ digital health, what forces them to share their voices.

“*Perhaps to see if there are possibilities that healthcare can take into account that there is a group of people for whom physical contact is still very important. If it is important for the patient or client, then chances are it is also important for recovery or the treatment plan*.” P6

### Financial reward

A financial incentive could be a motivation to become a panel member, however only one participant provided this as motivation. Most participants acknowledged that receiving a reward would be appreciated, but that this would not be the primary reason to participate. Conversely, some expressed the belief that they would not receive an award for participation, arguing that their intrinsic motivation (e.g., voice acknowledged and valued) was the primary reason for participation.

“*Because yes, if there was nothing in return [financial reward] I would be sitting at home now with no money. I do always try to help a little bit everywhere. But yes, I have limited time, so to speak. So that’s a big driver, though.”* P5

### Provide a meaningful contribution

Participants believed that they could contribute to the research panel and believed their input could enhance the accessibility and usability of digital health. They emphasized that their involvement would be instrumental in aligning digital health solutions to the specific needs and requirements of potential end users.

“*I don’t think I can influence that [digital health] very much, because it’s obviously something very big. Only, yes, I think at the moment several people think about a certain topic that you do get those blind spots, out of your research. That you can share things that the researcher has not thought of, but you as a citizen in practice, for example because you are in a certain age group or because you have little children [who experience certain issues].”* P10

## 2. Facilitators and barriers to panel participation

The facilitator theme pertains to the organisational responsibilities of panel managers and researchers, whereas the identified barriers represent challenges they must mitigate to optimise participants engagement and representation.

### Clear communication

Participants found it important that researchers let panel members feel comfortable and value their input. Researchers should therefore acknowledge their contribution and consider the provided input as valuable information. Participants also emphasized the importance of clear communication before a research take place. Specifically, they highlighted the need for information regarding the research topic, panel member expectations, and practical details such as date, time, and location.

“*Well, if I know what it’s about and how long it lasts. And that it is for example four times a year*.” P2

Opinions varied regarding communication between the panel organization and its members. Some emphasized the importance of maintaining contact during non-research periods, while others found it unnecessary. Overall, email communication was widely favoured, although for more complex research projects, a phone call was considered more effective.

“*That depends on what kind of survey it is, if just filling in a questionnaire, you can just do that via e-mail. Is it about participating in a test of a website or an application then yes, you have more questions, I think. And so then you would be better off using the phone for that.”* P16

### Feedback results after participation

The feedback of research results was considered very important by almost all participants. Participants were eager to hear about the outcomes of their input, even if it has had little or no impact. They remain highly interested in the results of the research they participated in. Because it reinforces the perception that their contributions are valued and actively utilized in the research process. Moreover, this feedback indirectly will sustain participants long-term enthusiasm for panel participation. There were only a few participants who did not consider it important to receive feedback on the research results, their motivation for participation lies in advancing research (external motivation), with intrinsic motivation playing a minor role.

“*I do think it’s very important that it’s appreciated. Because I also took part in a survey once, for example, about my condition and I actually got the feeling that I had had a kind of third-degree interrogation. I started feeling all guilty about my own illness. Well then, I thought never mind, I don’t need that anymore*.” P7

### Organisation of sessions

Regarding the organization of research sessions, responses varied significantly. Some participants highlighted the importance of having an enthusiastic researcher guiding the research session. Additionally, they emphasized the value of providing refreshments such as coffee, tea, and biscuits. Furthermore, several participants expressed a desire to meet fellow panel members in person, particularly during physical sessions like focus groups or co-creation sessions. For them, these gatherings served not only as research events but also as social interactions.

“*What may also be important, I have already participated in several projects, some offer tea, coffee, water. Sometimes research take a bit longer. I even had once, who started offering sandwiches because it took a bit longer: 2.5 hours. Yes, I know people do really appreciate that or at least a biscuit. I did see that people really liked that*.” P1

### Frequency of participation

During panel participation, most participants mentioned that participation in the panel should not be too burdensome. A majority of the participants expressed a preference for participating approximately four times a year, while also emphasizing the need for variety. For instance, they suggested a mix of activities, e.g., online interviews and interactive co-creation sessions in person.

### Barriers

The reasons deterring participants from engaging in a research panel were predominantly practical. Primary barriers were for example unreimbursed travel expenses, the time commitment and the travel distance. Participants may need to commute long distances, which could be particularly burdensome for those without easy access to (public) transportation. A number of participants residing outside the Amsterdam region exhibited a preference for online research participation.

An additional barrier was the perception that participation felt more as an obligation rather than a voluntary choice.

“*I don’t know if I would want to join, because I don’t know how many obligations it brings. If I just have to do something once, like now for an hour or so; no problem. But if I am obliged to do something 4 times a year on May, June, August and December, I feel my freedom is hampered. Then I think of yes, maybe I am on holiday. Maybe it’s not convenient, or then what? So in principle, I would like to join, provided it is not too squishy in obligations*.” P4

Only a minority of participants cited the excessive complexity demanded by the research project as a barrier to their participation in a panel. Low threshold participation options, for example offering telephone surveys instead of digital surveys, facilitates motivation.

“*The reason I wouldn’t do it, maybe if it [participation] gets a bit too difficult or if people would put too much pressure on you. But that’s more because I might not be able to handle that.”* P2

## 3. Prerequisites for panel participation

The theme ‘Prerequisites’ concerns the practical conditions that the panel must meet. Ensuring participation remained attractive for panel members to continue to participate actively for an extended period of time.

### Recruitment

#### Diversity in recruitment.

Several participants highlighted that the design of a flyer could be decisive for people to participate in a study. People should perceive a sense of appreciation upon registration and should feel that their opinions are valuable to the study. People should also get the feeling that they are welcome to join in the study. For example, participants were positive towards the design of the flyer (S2 File) for this interview study.

“*I myself don’t have that feeling of oh, it’s only for white people [pictograms of persons on flyer], because I have been in this country for so long, I already have this Dutch mentality. But you have some people, if they don’t see dark people [on the flyer], they think, oh it’s only for white.”* P2

“*Because as an older group, I’m 66, you feel less and less needed or people think you have no opinion at all. So yes, this[statement on flyer “give your opinion on research”] is meaningful. My opinion matters, it matters. Anyway, I thought it [text on flyer] was catchy.”* P3

Participants also indicated that it is important for an active approach strategy to include a diverse research team. Such as an older person and a person with a migration background. This diversity presented a comprehensive representation and enhanced the likelihood of a diverse group of individuals participating in the research.

#### Language and labels.

It proved difficult to describe inclusion criteria on a flyer without coming across as stigmatising. This study sought individuals in vulnerable positions, hence the terms “practical education level” and “age between 30 and 75 years” were used. However, participants found the term “practical education” ambiguous and somewhat associated with a lower level of education. The relevance of educational level was also questioned, especially by those who had completed their education decades ago. Another participant mentioned that you also have people from a trailer park, who have not had any education at all, and you exclude them by using “practical education” as the inclusion criteria. These labels could be stigmatizing and therefore appeared to be a barrier to recruiting individuals in a vulnerable position.

#### Locations for passive recruitment.

Participants’ preferences for flyer locations varied. Many favoured healthcare waiting rooms (i.e., at the general practitioner, physiotherapist, dentist or pharmacy), as they often read available health-related information there. Supermarkets were less preferred due to the focus on shopping. Some found religious institutions or libraries suitable, given the availability of time to consider participation in research.

“*Well, I think the General Practitioner because when people are waiting, which I do myself, I look around a bit and then I sometimes read what’s on those flyers that are hanging there, for example.”* P19

Social media was also chosen a good location to share the flyer, some of them had a personal preference for this medium. However, while many acknowledged the expansive reach of social media, but they did not prefer it as best location for themselves.

“*Social media, I don’t use that myself. That’s funny, because I think a lot of people would say that [as favourite location], but I’m not on social media myself, because I’m afraid of hacking and that my data will be misused.*” P4

In addition, some participants indicated that in neighbourhoods with low socio economic positions, people barely read. They indicated that distributing flyers is largely ineffective, as they are seldom read and fail to engage the target audience. Participants emphasized the importance of an active recruitment approach by researchers or apply snowball sampling. They suggested leveraging bridge builders - individuals with extensive networks who actively engage in connecting and recruiting others - to enhance outreach and participant efforts.

#### Locations for active recruitment.

Participants’ preferences for active recruitment locations varied. Shopping centres were generally deemed ineffective due to the hurried nature of shoppers. Opinions on markets were mixed, with weather conditions influencing receptivity. Community centres were viewed favourably due to their relaxed setting and the voluntary attendance of visitors.

“*At a shop, people are often in a hurry. Then not everyone is very relaxed. I think a community centre is like that, yes [....], where people sit quietly, drinking a cup of coffee*.” P9

Several participants underscored the necessity to explicitly state that the interaction pertains to a research project. It seemed crucial to avoid creating an impression of a sales pitch, as this could deter many individuals from engaging.

### Preferences regarding participation in studies

Participants had no pronounced preference for a specific study type. However, a majority of participants expressed readiness to participate in usability testing studies. They argued that their direct feedback facilitates rapid improvements in the design of a prototype, contrasting with the slower pace of, i.e., interview-based studies. Additionally, some participants highlighted the need to enhance user-friendliness in digital health, which they believe can be achieved through active participation in usability testing.

“*Yes I find a very nice one, because I very often ask myself the question, who tested this [website]? Because this doesn’t work. Yes, yes, I would like that very much*.” P7

### Rewarding preferences

Opinions on whether participants should receive rewards varied significantly. While no one considered rewards as the primary reason for research participation, many acknowledged that it could serve as a motivating factor. Participants emphasized that feedback on research results held greater value for them than a financial or material reward. However, some recognized that rewards might incentivize participation for other people in society.

Regarding preferred reward types, most participants expressed a desire for rewards that felt like gifts, enabling them to make special or additional purchase. A common suggestion was to provide gift cards after participating in research. Notably, approximately a quarter of participants favoured supermarket gift cards, which would enhance their grocery shopping budget and offer financial assistance. Almost no one favourited the fruit/grocery packages, hotel/restaurant cards, or the sports membership, due to practical reasons. Most participants preferred a gift card or a small gift (e.g., chocolates or flower seeds).

“*I think I like a book voucher or a gift voucher more, because going to the supermarket I do that anyway. And if I can buy a book once, or I can go somewhere once, which I might not do as easily otherwise, it feels more like a reward. This [supermarket voucher] feels more like keeping the household running.”* P4

We also asked participants whether they would opt for reward after research participation or periodically or by using a collection system. All participants agreed that post-research rewards would motivate them more and therefore prefer this type of rewarding. They argued that some panel members participate less frequently than others, and this would feel unfair with a periodic reward.

Participants found it challenging to specify an ideal reward amount. Some of them indicated that the minimal amount should be 20 euros, especially for physical meetings.

“*That also depends on how long the survey is. If it’s just a questionnaire, then I wouldn’t necessarily need/want a reward for that. But suppose it is a survey [on location] of 1-2 hours, maybe longer, then I would want to get a reward.”* P19

The majority of participants indicated that they would not expect an incentive for referring others through snowball sampling. Instead, when individuals within their social network expressed interest, they would voluntarily encourage them to join the panel.

## Discussion

### Main findings and interpretations

Given the complexity of recruiting and engaging a representative study population for (digital) health research, this study investigated whether individuals are willing to participate in a research panel focussed on digital health research. This study found that most participants were enthusiastic for engagement in such a panel. They appreciated having their voices heard and expressed a desire to help others facing similar challenges. Participants with personal experience using digital health tools often saw themselves as experts and were eager to share insights. However, it is important to understand participants enthusiasm in the light of the interview context, where detailed explanations and rapport-building likely boosted engagement. This implies that recruitment may benefit from similar dialogue, but also that our findings reflect a best-case recruitment scenario.

Those with higher digital health literacy appeared more likely to express motivations that sounded more intrinsic, while those with lower skills sometimes referred to personal or financial benefits. However, these differences were not consistent across all interviews and should therefore be interpreted cautiously.

Although participants framed their involvement as an opportunity to contribute to the future of digital health, some were motivated because of a perceived necessity to keep pace with ongoing advancements in the field of digital health. Rather than reflecting genuine enthusiasm, their engagement was therefore shaped by a desire to ensure future digital health services remain usable and aligned with their preferences. This highlights a form of engagement driven by apprehension that digital health services could otherwise become too complex or in-accessible. Key facilitators for recruitment and retention included timely feedback, appreciation of input, clear communication, and flexible participation options. Enthusiastic research leaders, appropriate rewards, and well-organized sessions - offering food, social interaction, and varied research formats - also supported engagement. Facilitators were consistent across literacy levels, though those with lower skills faced barriers when tasks felt too complex or cognitively demanding. Practical challenges such as travel and time commitment affected both groups.

Participants emphasized the importance of inclusive recruitment, suggesting flyer designs that convey appreciation and welcome diversity. Some preferred a diverse research team. All participants struggled with defining inclusion criteria clearly, terms like “practical education” were seen as ambiguous or exclusionary. They recommended using respectful and straightforward language to describe the desired study population. Effective recruitment strategies included direct engagement at community centres, placing flyers in general practitioner offices or pharmacies, and involving trusted key figures.

It may appear paradoxical that nearly all interview participants expressed enthusiasm about joining the panel, given that researchers typically face considerable challenges in recruiting a representative population. We hypothesize that this high level of enthusiasm can be attributed to the fact that, during the interview, ample time was devoted to explaining the nature of panel research, presenting examples of relevant projects, and clearly articulating the importance of their input. These efforts likely contributed to the participants’ increased motivation to engage.

### Integration with scientific knowledge

The motivations found in this study spanned intrinsic and extrinsic factors, as outlined in the self-determination theory [35]. The relative importance of these factors varied among participants, reflecting their diverse experiences and perspectives. Most participants were mainly intrinsic motivated in contributing insights, opinions and experiences to digital health research. Intrinsic motivation, according to the self-determination theory [35], can be increased by responding to three basic psychological needs: autonomy, competence, and relatedness. This aligns with what participants in this study consider important, namely they valued the opportunity to share their perspectives and appreciated researchers’ recognition of their input (*autonomy*). Many participants expressed a desire to contribute to society and assist others facing similar challenges (*relatedness*), to influence developments and help improve to accessibility of digital health tools by participating in research (*competence*). Only a few participants had an extrinsic motivation for participation, including an altruistic attitude or being motivated due to financial incentives. A study by van Leersum et al. [36] about the involvement of diabetes patients in digital health research, also found that most people were intrinsic motivated, indicating that they found it important to improve digital health, aiming to make it more usable and accessible for themselves and peers.

Our findings also align with research on “hard-to-reach” populations [37]. They emphasized the importance of flexible participation options and recognizing the cognitive and physical demands placed on participations. Researchers should be aware that participants feel comfortable and qualified for participation, as outlined in a study among younger underserved populations by Denford et al. [38], and a study by Goedhart et al. [14] among individuals living in vulnerable circumstances. Both argue that this helps to keep individuals engaged for long term participation. While some studies highlights barriers, for example a study about e-/mHealth argues that power dynamics may hinder participants’ engagement, such as patients not being seen as equal partners, conflicts of interest among stakeholders, or lack of decision-making power [26]. However these factors are not found in this study. Power dynamics may not have emerged strongly in interviews about anticipated participation and should therefore be explored during real panel operation.

Prerequisites that emerged from this study contains the active recruitment of individuals in a vulnerable position, encompassing visiting community centres or public libraries. These findings are supported by prior work [14,15,39]. Participant’s mixed view on rewards also echoed van Leersums study [36] who suggested that researchers should consult participants during recruitment about preferred incentives, to make participation more personally attractive.

### Strengths and limitations

Strengths of this study included the inclusion of a diverse target group, considering diversity in many demographics including, educational level, employment status, socio economic position, health-, and digital health literacy. Part of the outcomes of this study regarding recruitment strategies (e.g., in community centres) were already applied in this study. Participants confirmed that these methods were effective for including a representative population. Secondly, we provided participants with the option to conduct the interview digitally or in person, either at a designated location or at participants’ homes, thereby lowering the participation threshold.

However, this study is also subject to several limitations. Firstly, we used a (non-validated) shortened version of the eHEALS to measure digital health literacy. This may potentially impact the reliability of the classification of participant’s literacy. Additionally, digital health literacy was solely measured using a self-reported instrument, and practical experience suggests that participants overestimate their competencies. It is also remarkable that some participants reported never having used digital health services, yet still scored high on the DHL questionnaire. Second, the participants who participated in the online interview, completed the NVS-D assessment for health literacy at home. It is conceivable that participants may have looked up answers online or collaborated with others during this process, potentially compromising the reliability of their health literacy assessment. Third, the use of two interviewers and two data collection waves may have introduced subtle variation, although we aimed to ensure consistency and no clear thematic differences emerged. Fourth, it is also possible that social desirability bias was present during the interviews. Especially when we asked participants what they could motivate to become panel member, they could have given socially desirable responses. This study shows that people seem very intrinsic motivated, however these results can be biased since they were also motivated to participate in this interview study. Fifth, because our sampling relied on community centre recruitment and recontacting previous participants, the study likely included people already comfortable with research. This may have increased reported willingness to join a panel and influenced the themes, while perspectives of more hesitant or harder- to-reach individuals may be underrepresented. Sixth, our sample also included substantially fewer males than females, which may have influenced the findings. Although we did not observe clear differences in attitudes by sex, the small number of male participants limits our ability to draw firm conclusions about their motivations or barriers. Seventh, the Dutch context, Dutch language requirement, and recruitment in specific community locations also limit transferability to other regions or communities with different linguistic or cultural backgrounds. Lastly, the average age of 54 (oldest participant was 76) limits the transferability of our findings to specific age related perspectives or experiences.

### Implications for research and practice

**Fig 2:**
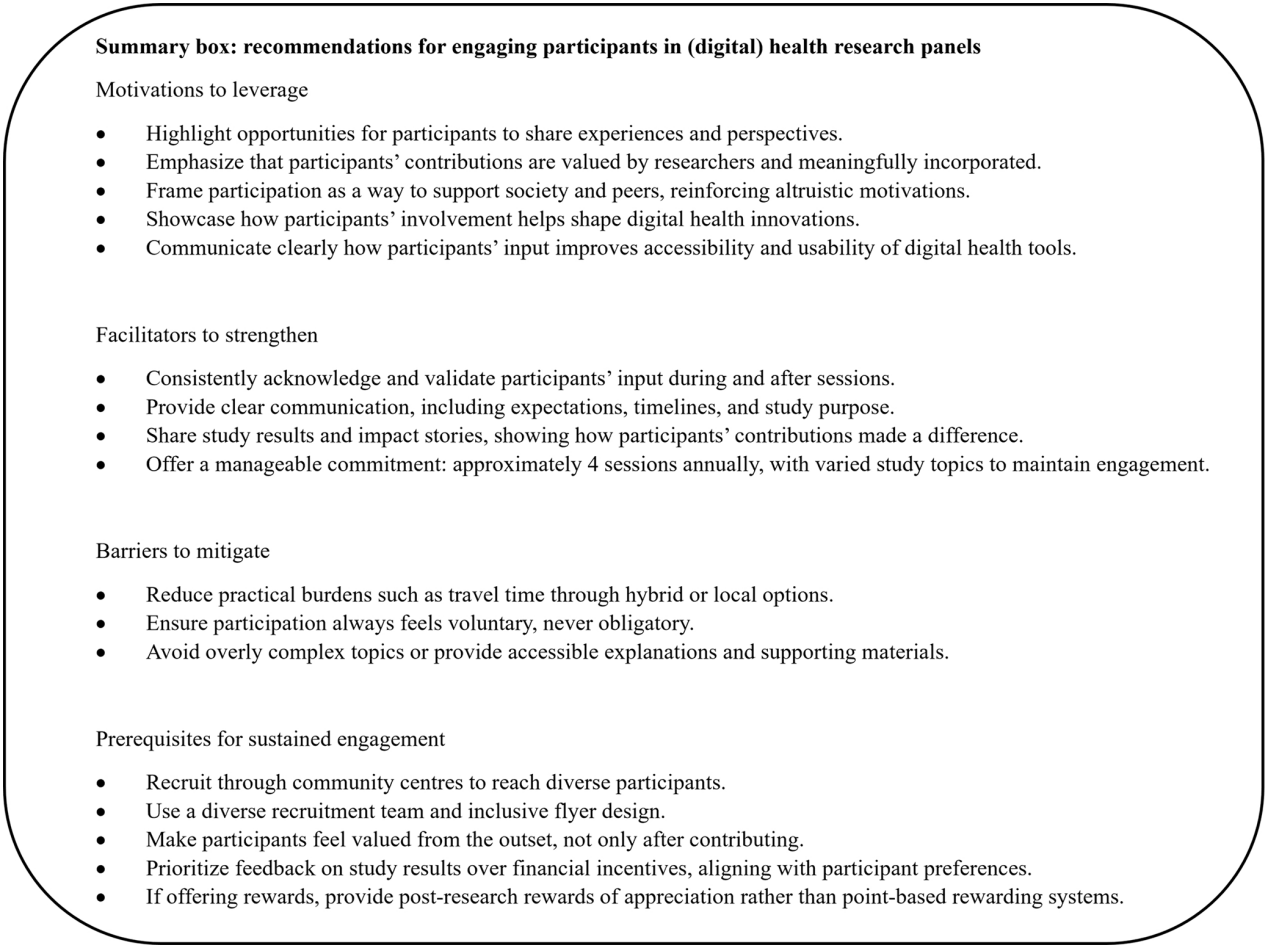
Practical recommendations.

The findings of this study offer insights for both panel managers and researchers working with individuals in vulnerable positions (see also Fig 2). Although the study focussed on digital health, the implications are likely applicable across other domains that engage similar populations. Many of the motivational factors we identified, such as wanting to help others, desire for societal contribution, or having a voice acknowledged, but also recruitment strategies, rewarding’s or practical barriers are not specific to digital health contexts.

Based on this research we recommend researchers and panel managers to recognize the heterogeneity in participants’ motivations for engaging in (panel) research. Incorporating motivational profiling during recruitment. Practical examples of brief profiling questions include: *“Why are you interested in participating?”; “What would make participation easier for you?”; “Which modes of participation do you prefer (in‑person, phone, video)?* Motivational profiling can help tailor recruitment strategies, align expectations, and foster a more committed and relevant participant base. Addressing participation barriers is essential for inclusivity. Practical adjustments, such as offering remote participation to reduce travel burdens, scheduling sessions outside standard working hours, and compensating participants fairly, can significantly enhance engagement. For participants with low digital health literacy, provide concrete non‑digital alternatives (telephone interviews, mailed paper surveys with prepaid return envelopes, and in‑person sessions at accessible locations).

In addition, clear and empathetic communication throughout the research process is critical. This includes setting transparent expectations, ensuring participants feel respected and valued, and providing timely feedback, for instance short summaries of study results, infographics on project milestones, or short videos explaining how their input was used. These mechanisms for feedback and demonstrating the generated impact, can strengthen trust and motivation, especially among those in vulnerable circumstances. By integrating the provided strategies, research (panels) can become more inclusive, responsive and effective in capturing diverse perspectives, ultimately enhancing the quality and applicability of research outcomes.

## Conclusions

In conclusion, participants in this study expressed a high level of interest in participating in a research panel focusing on digital health. Their motivation was primarily intrinsic, driven by factors such as having their voices acknowledged, societal contributions, and the desire to influence digital health developments. For sustainable participation, they emphasized the importance of clear communication about research projects they are involved in and providing feedback on study results. Practical barriers such as time commitment and travel distance, together with psychological barriers like perceived obligation, may hinder participation. By acknowledging and addressing these barriers, researchers can create a more inclusive and supportive environment for potential participants. Importantly, the factors influencing engagement in digital health research are highly relevant to other health related research projects. Many of the same motivational drivers, such as the desire to contribute to health advancements and improve accessibility, also apply to participation in general health related studies. Understanding these shared dynamics can help researchers develop more effective strategies for fostering sustained participation and ensuring that individuals in vulnerable positions are meaningfully represented in the development and evaluation of (digital) health innovations.

## Supporting information

S1 File Interview Guide. (DOCX)

S2 File Flyer. (JPG)

S3 File Interview Cards. (PDF)
