# Supplementary material for: Engaging individuals in digital health research panels: A qualitative study including participants in vulnerable positions
Source: PLOS Digit Health. 2026 May 22;5(5):e0001443. doi: 10.1371/journal.pdig.0001443 (PMC13196978; doi:10.1371/journal.pdig.0001443)
Supplement: S1 File — (DOCX) [file pdig.0001443.s001.docx]

# Interviewguide

**Introduction**

Thank you for agreeing to participate in this study. In this part of the study, we will ask you a number of questions (this is called an interview). We are interested in your answers, as they will help us in our research.

We will ask for your opinion or experience, so there are no wrong answers!

The interview is about setting up a panel. A panel is a fixed group of people who participate in a survey several times a year. The theme of the surveys is digital healthcare. Digital healthcare is a very broad topic, e.g. video calls with your doctor, a website such as thuisarts.nl, measuring your blood sugar levels at home, etc.

In this interview, we will ask you what you think about participating in such a panel. Reasons for not participating are also important for us to know.

So, it's all about what you think!

The interview will take approximately 40 minutes, after which you will fill out a questionnaire with your details. We can also fill this out together, if you prefer.

- Signing the informed consent form

We would like to record this interview (audio). Is that okay with you?

- Yes, start recording.

Structure of the interview

We will ask questions about the following six topics:

[0] Introductory questions, getting acquainted, what you already know about research

[1] What motivates you to participate in a panel

[2] How can we best approach you for a study

[3] How can we best stay in touch with you + how will you stay connected

[4] How would you like to be rewarded for being on the panel and/or participating in studies

[5] Practical matters, where would a study best take place, etc.

Interview questions

**[0] Introductory questions [0-5 minutes]**

0a. Do you use digital healthcare? What comes to mind when you think of digital healthcare? Do you ever visit thuisarts.nl when you are experiencing health issues?

- If yes: what is your experience with digital healthcare? Do you find that digital healthcare is beneficial? In what way?

0b. What comes to mind when you think of scientific research? Have you ever participated in scientific research? What do you already know about it?

- If yes: What are your experiences with research?

0c. How do you envision such a panel/research group?

**[1] What motivates you to participate in a panel [10-15 minutes]**

1a. Can you tell us why you are participating in a study (motivation)? You are now participating in this interview. Why?

Researchers are increasingly asking people to participate in research.

1b. What do you think about researchers increasingly asking citizens to participate in research?

1c. Would you be willing to participate in multiple studies (non-physical) for an extended period of time (several years)? What makes you enthusiastic?

o Do you think it is important that digital healthcare is understandable to everyone? If so, is this a reason why you would like to participate in a panel?

1d. How could we convince you to participate in our studies?

- How would you like to be supported?

- What would you need in order to participate?

1e. What do you expect to learn or achieve by participating in multiple research projects on digital healthcare?

1f. Are there other reasons that have not yet been mentioned?

1g. Why would you not want to participate in a panel? What is stopping you?

Summarize what the respondent has said. I understand that you ... and this ... is that correct?

**[2] How can we best approach you for a survey [5-10 minutes]**

2a. Present recruitment strategies to participants

Flyer:

- Circle three places where you think the flyer would be most noticeable Flyer slide 1

- If you see information on a flyer, does it encourage you to contact us to participate in a survey?

Active approach:

- How can we best get in touch with you? Where? Active approach slide 2

Difference between passive and active approaches:

- What do you think works better: a flyer in, for example, a supermarket, or being asked by a researcher at, for example, a community center? Or something else?

2b. Do you have any tips on how we can ask people to participate in research?

- Should we do this ourselves OR is it better to have others do it? E.g. people who already have contact with the target group

- Which organizations could we collaborate with? For example: community centers, churches, mosques, food banks, etc.

2d. What do you think when we say we are looking for people with practical training? [Show flyer]

- What do you think when we say we are looking for people who have difficulty understanding health information? Do you find this stigmatizing? Or clear?

Summarize what the respondent has said. I understand that you ... and this ... is that correct?

**[3] How can we best stay in touch with you + how can we keep you engaged [10 minutes]**

3a. How can we ensure that you remain in our panel for a longer period of time? And thus participate in various surveys for a longer period of time.

3b. How can we keep you enthusiastic about participating in our panel?

- Rewards A. after each survey or B. annually

- Feedback on the survey results

o If so, how: video, website, email, WhatsApp

3c. What do you think should be done with the results of a survey? Published in a local newspaper?

- Ask follow-up questions

3d. How should we stay in touch with you?

3e. Do you have any tips to ensure that people continue to participate and remain involved?

Summarize what the respondent has said. I understand that you ... and this ... is that correct?

**[4] How would you like to be rewarded for participating in the panel and/or surveys?**

4a. How would you like to be rewarded (gift) after participating in a survey?

4b. The municipality of Amsterdam wants to promote healthy living. What do you think of the following rewards?

- First ask people what they think of this

- Participants should then rank them in order of “importance.”

4d. What do you think of a points system, where you receive a number of points after each survey you participate in? If you have participated multiple times, can you receive an extra reward?

- What do you think of snowball sampling? For example, you are in the panel and you refer neighbors/family members or friends. What do you think of this method?

Summarize what the respondent has said. I understand that you ... and this ... is that correct?

**[5] Practical matters, where would be the best place to conduct the research, etc. [5 minutes]**

Now for the last few questions:

5a. What kind of research would you be willing to participate in? Type of research

• Questionnaires (you can do this at home on your computer)

• Interviews (can also be done by telephone)

• Focus group

• Co-creation

• Testing an app or website

5b. Are you willing to travel for the research (to the AMC)? Or would you prefer to do it at home, in a community center, library, or other location in your neighborhood? Some research can also be done by phone or video call.

Summarize what the respondent has said. I understand that you ... and this ... is that correct?

**Finally**

Do you know anyone else who would like to participate in the interview?

Thank the participant for their participation

Tell them about the possible follow-up to the study and what we will do with these results
