# Supplementary figures and images for: Engaging individuals in digital health research panels: A qualitative study including participants in vulnerable positions
Source: PLOS Digit Health. 2026 May 22;5(5):e0001443. doi: 10.1371/journal.pdig.0001443 (PMC13196978; doi:10.1371/journal.pdig.0001443)

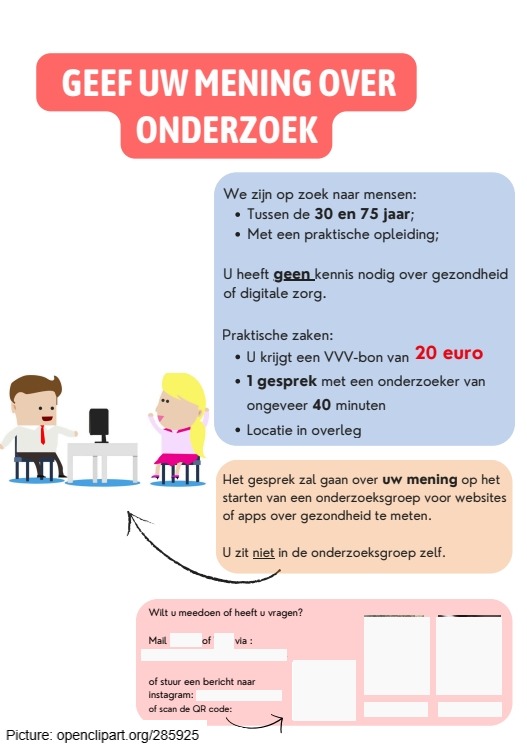

Supplement: S2 File — (JPG) [file pdig.0001443.s002.JPG]
