# Supplementary material for: Engaging individuals in digital health research panels: A qualitative study including participants in vulnerable positions
Source: PLOS Digit Health. 2026 May 22;5(5):e0001443. doi: 10.1371/journal.pdig.0001443 (PMC13196978; doi:10.1371/journal.pdig.0001443)
Supplement: S3 File — (PDF) [file pdig.0001443.s003.pdf]

Flyer :

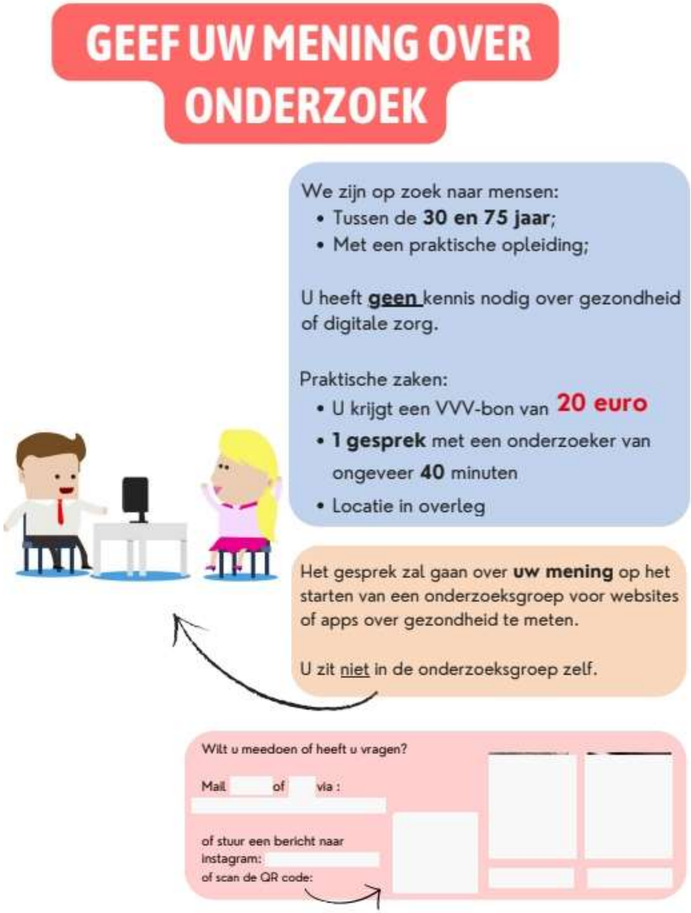

Where would the flyer be most noticeable to you?

- ☐ Food bank
- ☐ Supermarket
- ☐ Church/ Mosque
- ☐ Hairdresser
- ☐ Library
- ☐ General practioner, Physiotherapy (Waiting room)
- ☐ Social media (Facebook or Instagram)
- ☐ Local restaurant
- ☐ Other: ____________

Picture: openclipart.org/285925

In which place can we best get in touch with you?

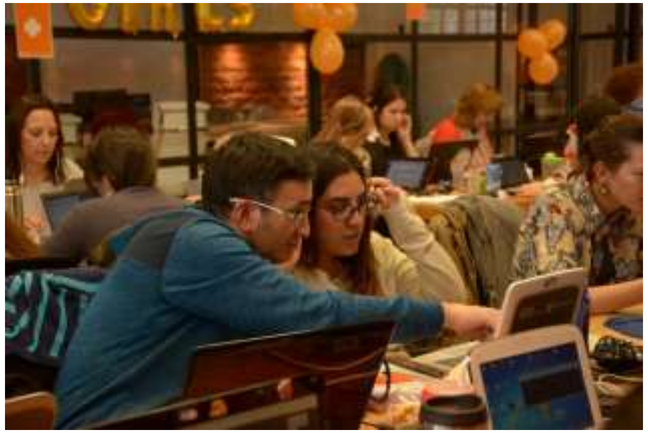

☐ Having a chat in a community centre

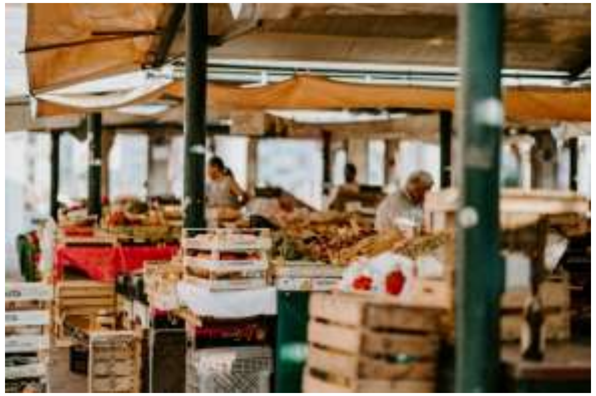

☐ Having a chat on the market

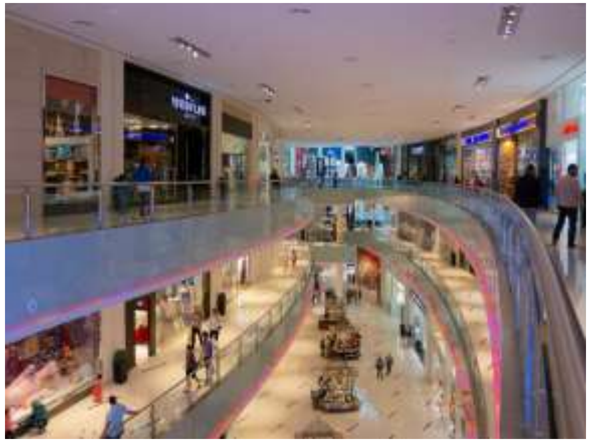

☐ Having a chat in a local shop

☐ Other location: _____________

Community centre: Photo by fran innocenti on Unsplash

Market: Photo by Annie Spratt on Unsplash

Local shop: Photo by mostafa meraji on Unsplash

A montly fruit or vegetable box

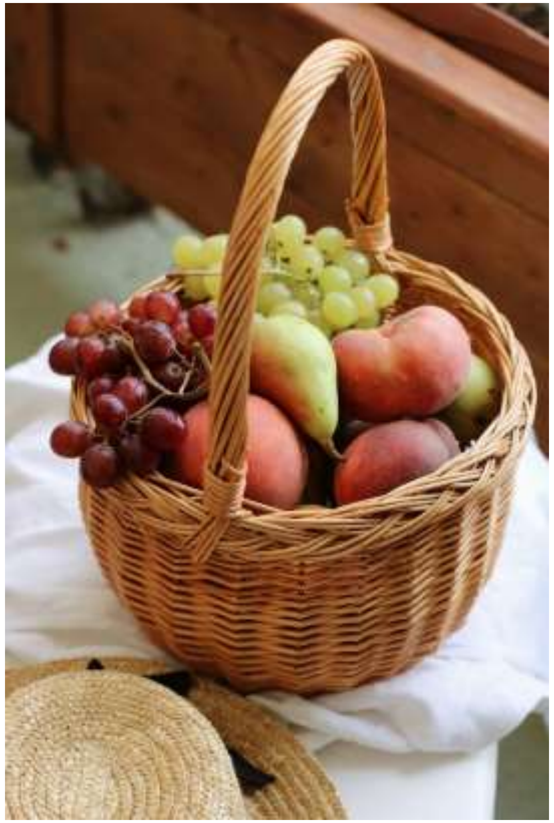

Photo by Nataliya Melnychuk on Unsplash

A montly supermarket giftcard

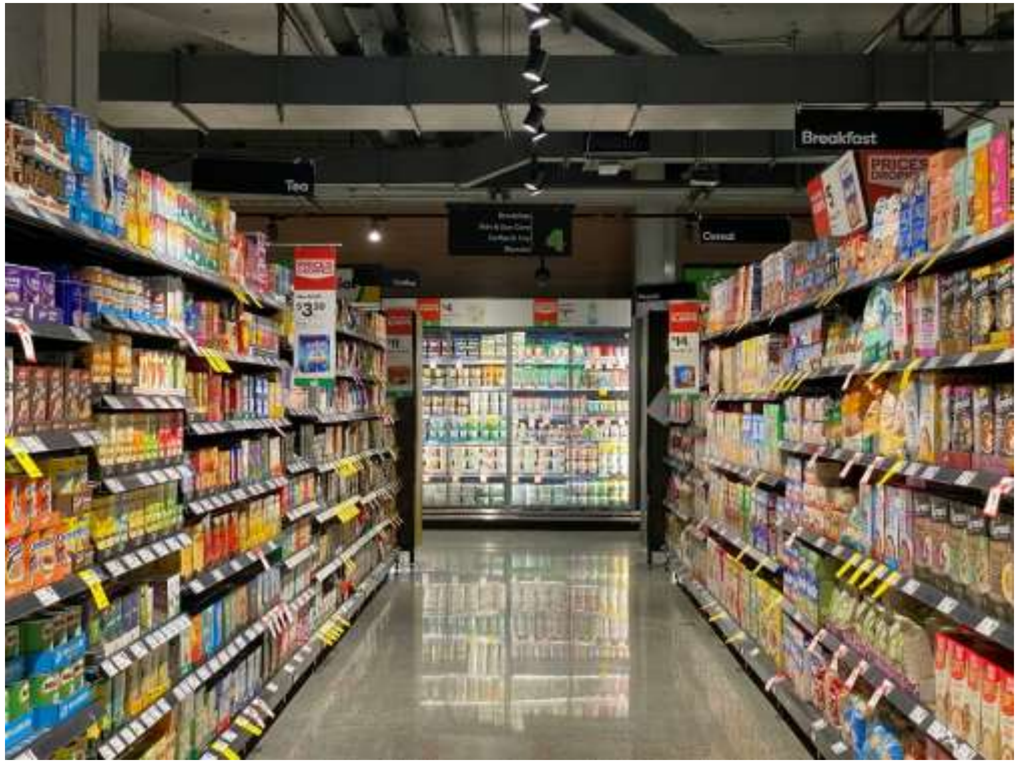

Photo by Franki Chamaki on Unsplash

Dinner and hotel cheque

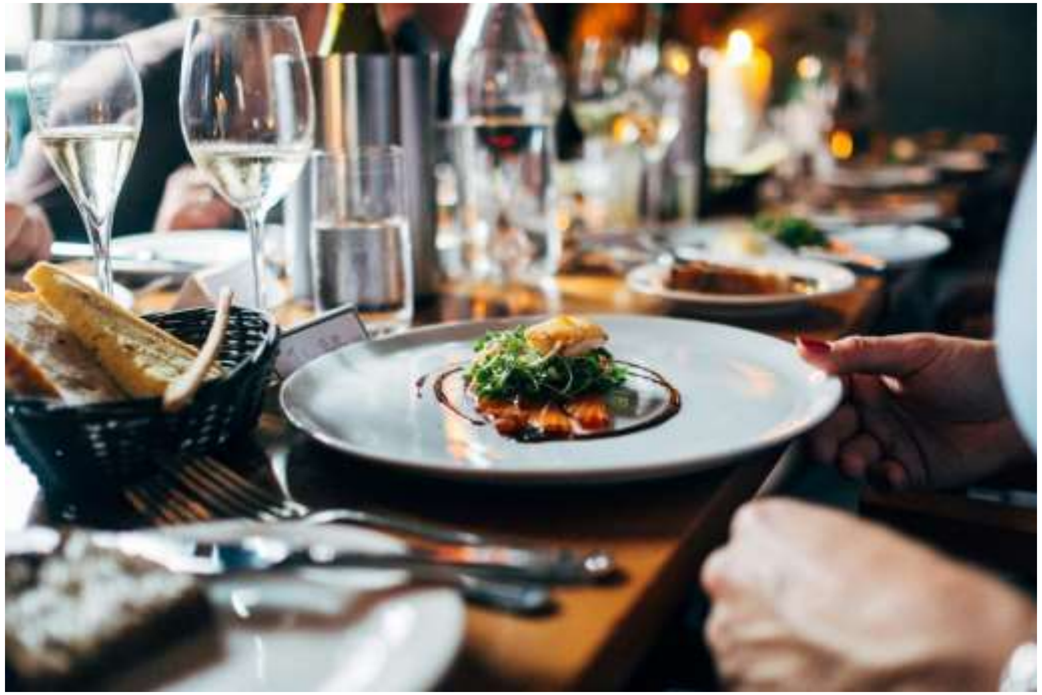

Photo by Jay Wennington on Unsplash

6 months free sport membership

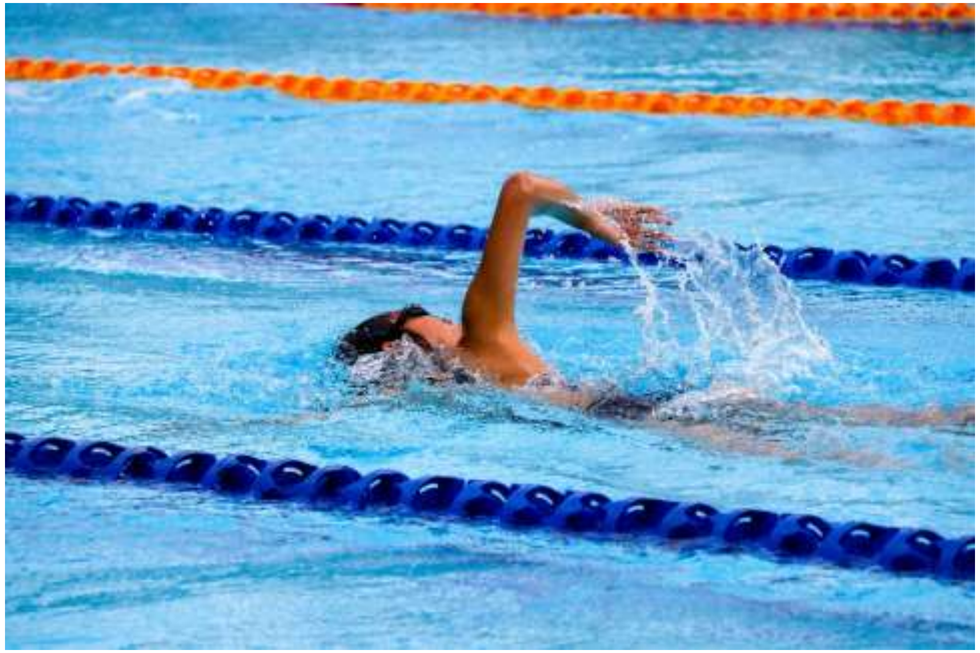

Photo by Marcus Ng on Unsplash

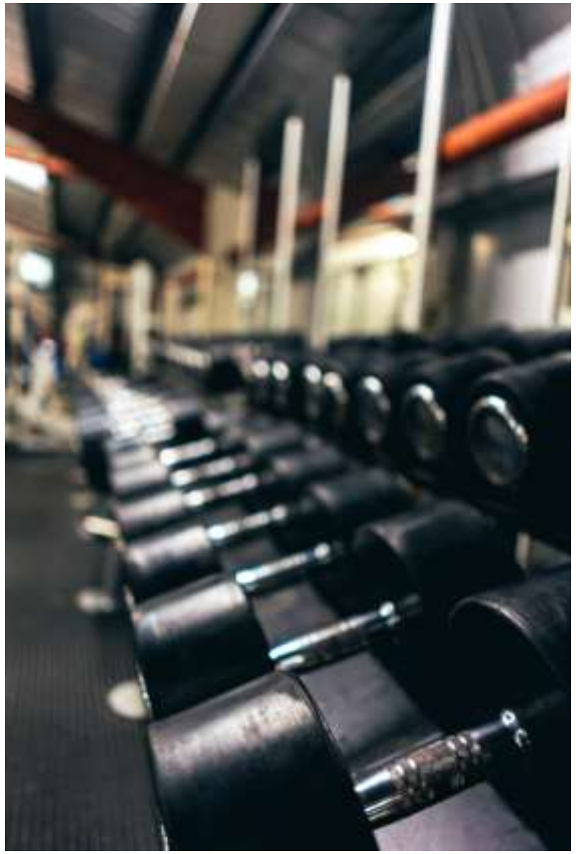

Photo by Samuel Girven on Unsplash

Enjoyable outing with other members of the panel

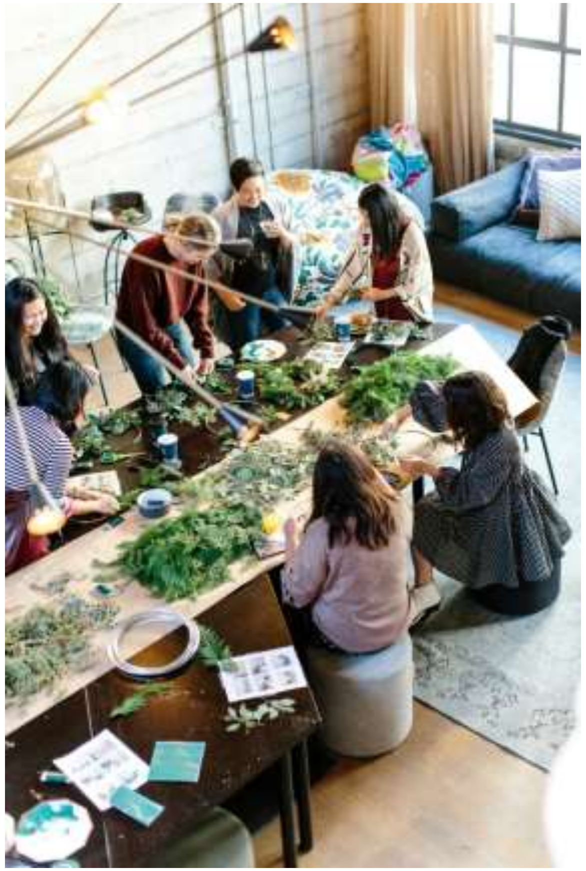

Photo by Hillary Ungson on Unsplash

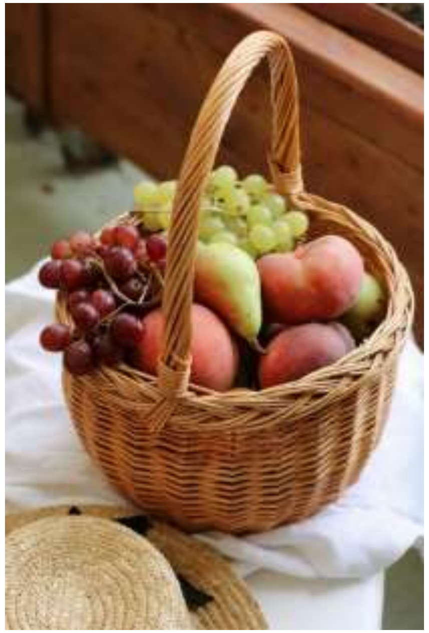

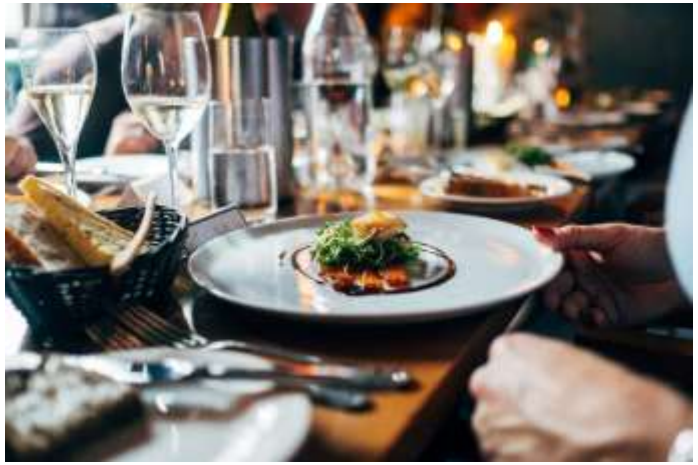

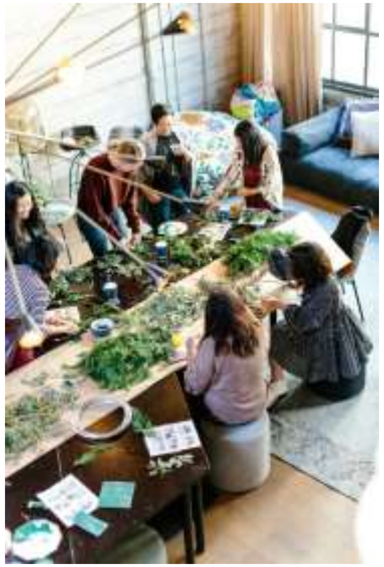

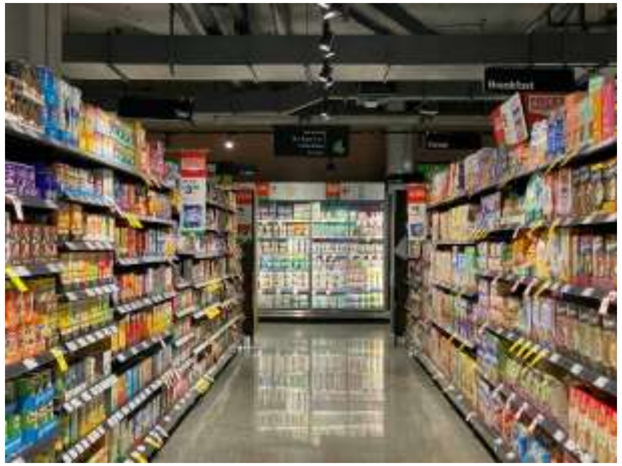

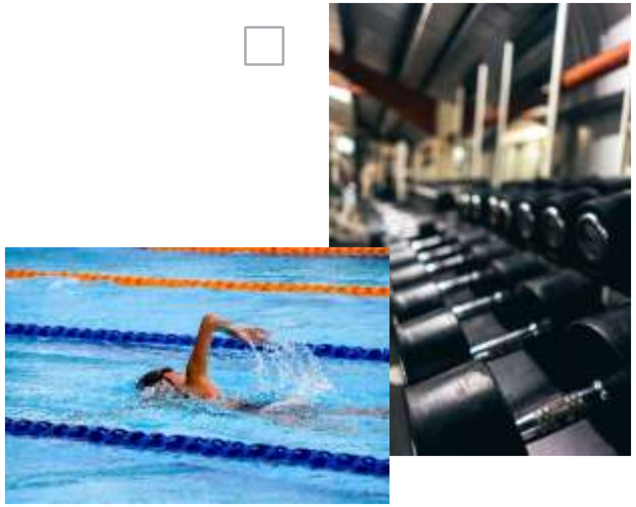

Other:

Rewarding system

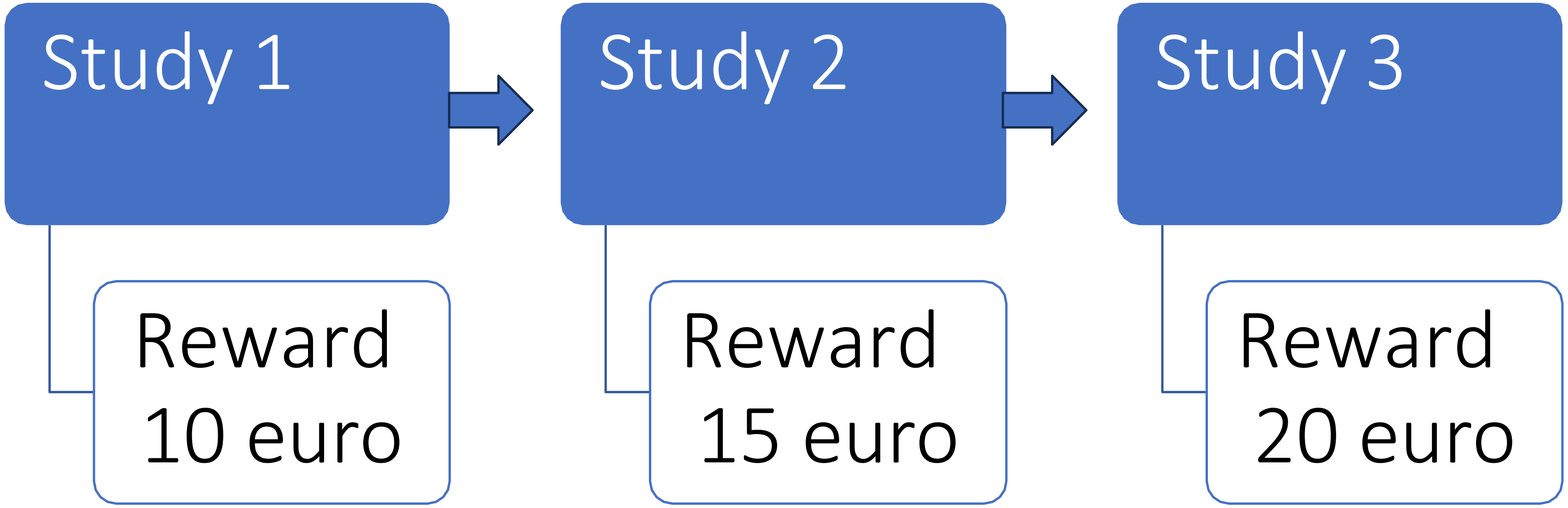

Reward for introducing a new panel member

I would like to participate in this study:

☐ Interview (chat with a researchers)

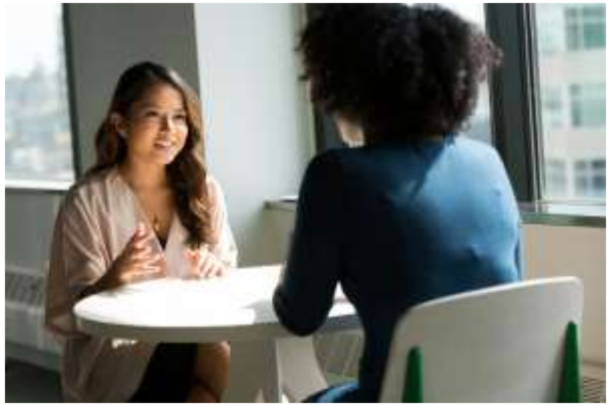

Photo by Christina @ wocintechchat.com M on Unsplash

☐ Filling in surveys

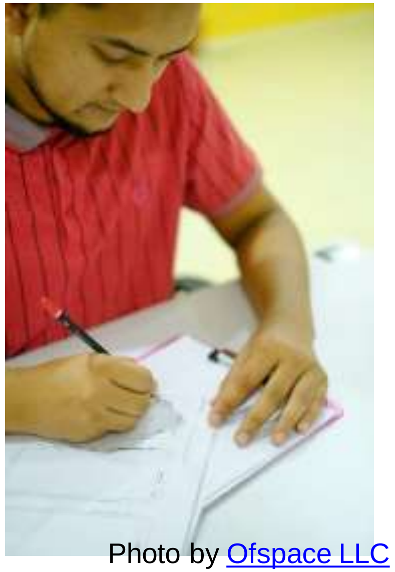

Photo by Ofspace LLC on Unsplash

☐ Telephone interview

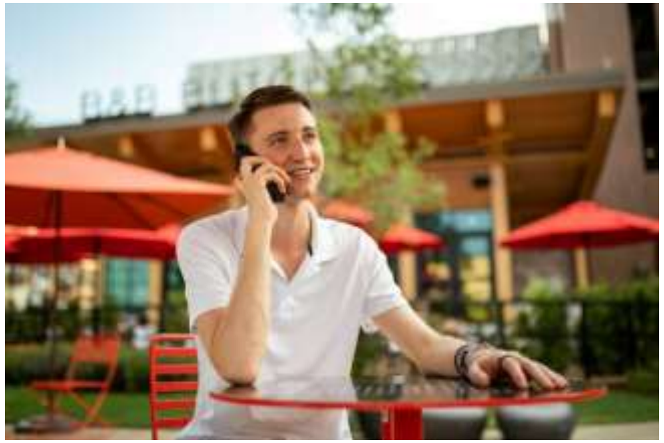

Photo by Chase Chappell on Unsplash

I would like to participate in this study:

☐ Co-create a website or application

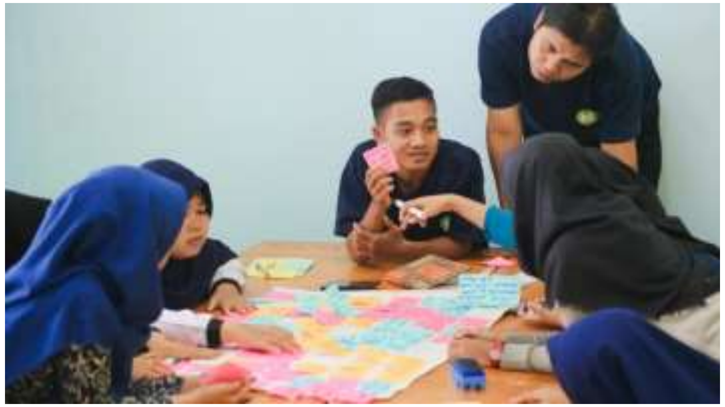

Photo by Zainul Yasni on Unsplash

☐ Chat with other people and a researcher

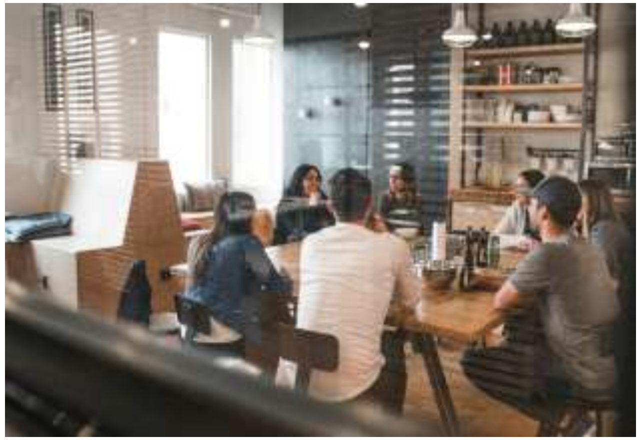

Photo by Redd Francisco on Unsplash

☐ Test a website or application

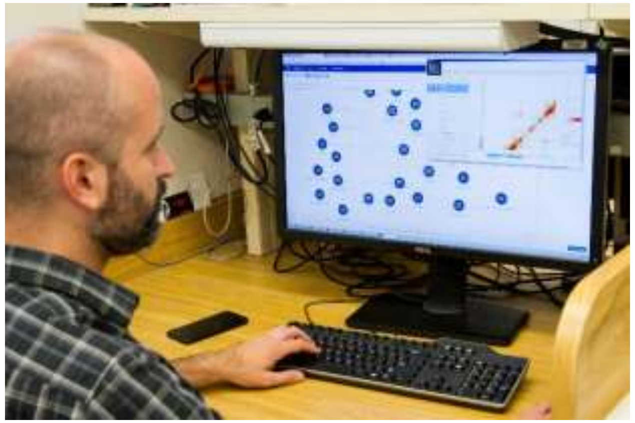

Photo by National Cancer Institute on Unsplash
